# Superstition in Surgery: A Population-Based Cohort Study to Assess the Association Between Surgery on Friday the 13th and Postoperative Outcomes

**DOI:** 10.1097/AS9.0000000000000375

**Published:** 2024-02-12

**Authors:** Sanjana Ranganathan, Carlos Riveros, Michael Geng, Courtney Chang, Yusuke Tsugawa, Bheeshma Ravi, Zachary Melchiode, Siqi Hu, Kathleen Kobashi, Brian J. Miles, Zachary Klaassen, Avery Nathens, Natalie Coburn, Allan S. Detsky, Angela Jerath, Christopher J. D. Wallis, Raj Satkunasivam

**Affiliations:** From the * Department of Urology, Houston Methodist Hospital, Houston, TX; † Division of General Internal Medicine and Health Services Research, David Geffen School of Medicine at UCLA, Los Angeles, CA; ‡ Department of Health Policy and Management, UCLA Fielding School of Public Health, Los Angeles, CA; § Division of Orthopedic Surgery, Department of Surgery, Sunnybrook Health Sciences Center, Toronto, Ontario, Canada; ‖ Division of Orthopedic Surgery, Department of Surgery, University of Toronto, Toronto, Ontario, Canada; ¶ Division of Urology, Medical College of Georgia, Augusta University, Augusta, GA; # Division of Surgery, Department of Surgery, Sunnybrook Health Sciences Centre, University of Toronto, Ontario, Canada; ** Department of Medicine, Mount Sinai Hospital and University Health Network, Toronto, Ontario, Canada; †† Institute for Health Policy, Management and Evaluation, University of Toronto, Toronto, Ontario, Canada; ‡‡ Department of Medicine, University of Toronto, Toronto, Ontario, Canada; §§ Department of Anesthesia, Sunnybrook Health Sciences Center, Toronto, Ontario, Canada; ‖‖ Division of Urology, Department of Surgery, University of Toronto, Toronto, Ontario, Canada; ¶¶ Division of Urology, Department of Surgery, Mount Sinai Hospital, Toronto, Ontario, Canada; ## Department of Surgical Oncology, University Health Network, Toronto, Ontario, Canada.

**Keywords:** cultural beliefs, Friday the 13th, postoperative outcomes, surgery, superstitions

## Abstract

**Objective:**

We sought to examine whether the outcomes of patients who receive a surgical procedure on Friday the 13th differ from patients who receive surgery on flanking Fridays.

**Background:**

Numerous studies have demonstrated that increased anxiety from the provider or patient around the time of surgery can lead to worse outcomes. Superstitious patients often express significant concern and anxiety when undergoing a surgical procedure on Friday the 13th.

**Methods:**

A retrospective, population-based cohort study of 19,747 adults undergoing 1 of 25 common surgical procedures on Friday the 13th or flanking control Fridays (Friday the 6th and Friday the 20th) between January 1, 2007, and December 31, 2019, with 1 year of follow-up. The main outcomes included death, readmission, and complications at 30 days (short-term), 90 days (intermediate-term), and 1 year (long-term).

**Results:**

A total of 7,349 (37.2%) underwent surgery on Friday the 13th, and 12,398 (62.8%) underwent surgery on a flanking Friday during the study period. Patient characteristics were similar between the 2 groups. We found no evidence that patients receiving surgery on Friday the 13th group were more likely to experience the composite primary outcome at 30 days [adjusted odds ratio (aOR) = 1.02 (95% CI = 0.94–1.09)], 90 days [aOR = 0.97 (95% CI = 0.90–1.04)], and 1 year [aOR = 0.99 (95% CI = 0.94–1.04)] after surgery.

**Conclusion:**

Patients receiving surgery on Friday the 13th do not appear to fare worse than those treated on ordinary Fridays with respect to the composite outcome.

## Abstract 2

Mini abstract

Superstitious patients often express significant concern and anxiety when undergoing a surgical procedure on Friday the 13th. In our retrospective cohort, we found no evidence to suggest that patients undergoing surgery on Friday the 13th were more likely to experience death, or short- and long-term complications.

## INTRODUCTION

The belief that Friday the 13th is a doomed day is a popular superstition that has found its way into many domains of culture. *Paraskevidekatriaphobia* is a term used to describe the fear of the date, with a history that dates back many centuries.^1^ The origins of the lore can be traced to the Bible: Jesus Christ was believed to have been crucified on a Friday; Judas was the 13th apostle at the Last Supper who betrayed Christ’s trust; and Cain killed his brother Abel on Friday the 13th.^2^ Consequently, there are many manifestations of this superstition in daily life. Airports often do not have a gate 13 and many hotels and hospitals skip the 13th floor. Consumer behavior appears to be influenced, with lower stock returns on the date,^3^ and traffic accidents have been shown to be more likely on Friday the 13th.^4,5^

Superstition aside, perspectives and attitudes can have profound impacts on behavior. Providers who are stressed or anxious tend to make more errors, which can negatively impact patient outcomes.^6,7^ Likewise, patient concerns often creep their way into medical practice. Adolescents who are more anxious about postoperative pain tend to subsequently require more pain medication^8^ and adults with negative perceptions about aging have slower walking speeds later in life, underlying ways in which beliefs predict physical decline.^9^ The psychological concept of a self-fulfilling prophecy, wherein ideologies influence behavior and consequently impact outcomes, could be applied to Friday the 13th superstition. It stands to reason that fear of Friday the 13th could be enough to provoke anxiety and worry among providers and patients, leading to poor outcomes.

Within the field of medicine, there has been exploration into whether Friday the 13th increases the likelihood of harm and poor outcomes, with mixed findings. Although there are no differences in mortality after acute coronary syndrome on Friday the 13th^10^ nor in the number of emergency room visits,^11^ there is evidence of increased rates of penetrating trauma^11^ and hospital admissions due to transportation-related injuries.^12^ Limited evaluations of surgical outcomes have been performed. Examining tonsillectomy and the frequency of emergency operations have not revealed meaningful differences on Friday the 13th.^13,14^

Is Friday the 13th a safe day to go to the operating room? We sought to assess whether surgical outcomes differed for patients treated on Friday the 13th by assessing a large, population-based cohort encompassing all surgical specialties, both emergent and nonemergent surgeries, and considering both short-term (30-day) and long-term (90-day and 1-year) outcomes.

## METHODS

### Study Setting and Design

We conducted a population-based, retrospective cohort study of adults undergoing common surgical procedures in Ontario, Canada, between January 1, 2007, and December 31, 2019. In Ontario, residents have access to a provincial universal healthcare plan delivered by a single government payer, the Ontario Health Insurance Program (OHIP). We utilized the OHIP database to select procedures across a variety of subspecialties, including cardiothoracic surgery, general surgery, neurosurgery, obstetrics and gynecology, orthopedic surgery, otolaryngology, plastic surgery, thoracic surgery, urology, and vascular surgery.^15–17^ This study was performed in accordance with Strengthening the Reporting of Observational Studies in Epidemiology guidelines^18^ and REporting of studies Conducted using Observational Routinely-collected health Data statement.^19^ The study protocol was approved by the Mount Sinai Hospital Research Ethics Board.

### Data Sources

We retrieved data from multiple healthcare databases which are available from the Institute for Clinical Sciences (IC/ES). Procedural claims are recorded in the OHIP database, which was used to gather information regarding primary surgical procedures and complications. The Canadian Institute for Health Information, Discharge Abstract Database, and Same Day Surgery databases were utilized to obtain diagnostic, procedural, and discharge data. Patient demographic information was accessed via the Registered Persons Database. Physician demographic information was accessed via the IC/ES Physician Database.

### Cohort Derivation

**FIGURE 1.**
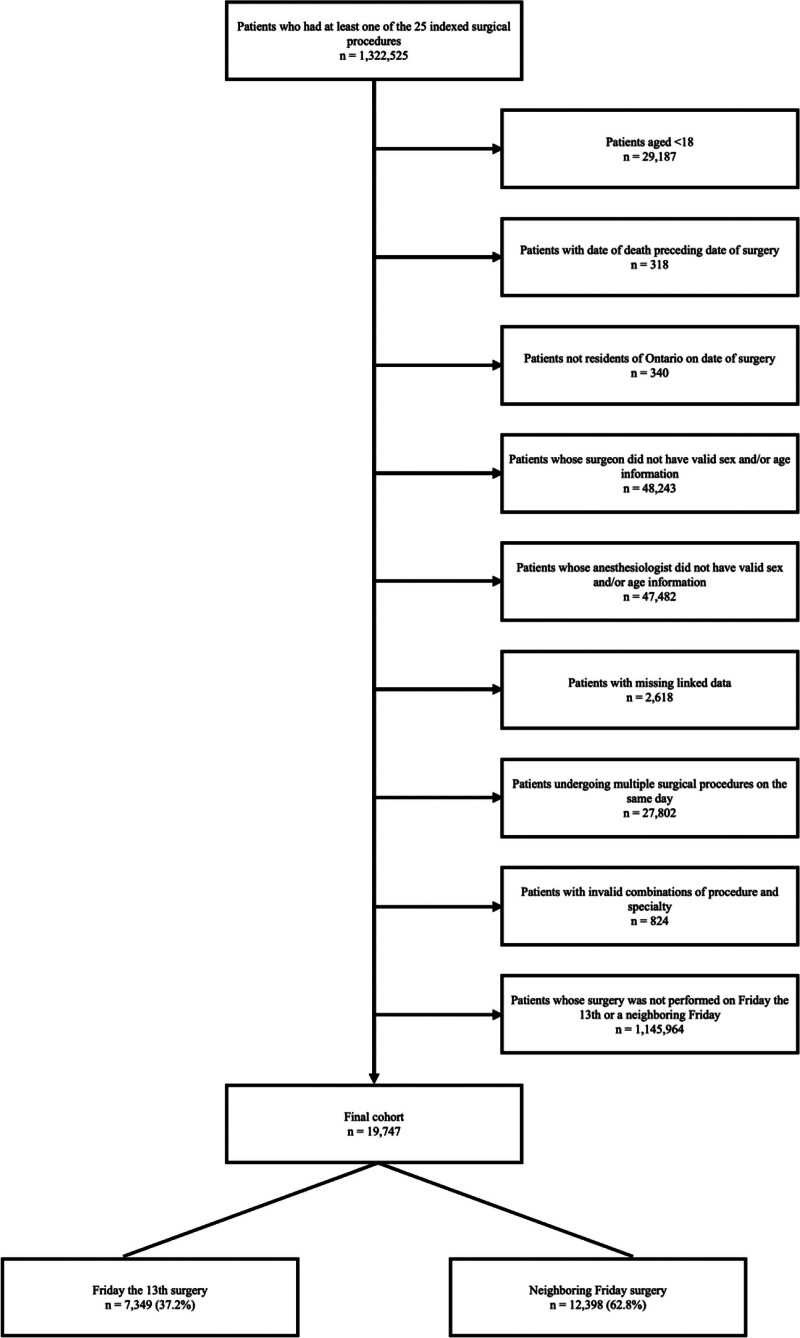
CONSORT flow diagram.

We selected patients who had undergone any of the 25 procedures of interest (Supplemental Table 2, http://links.lww.com/AOSO/A289) during the study period (Fig. 1). We excluded patients who were younger than 18 years old at the time of surgery, patients with multiple procedures on the same day, and patients with invalid combinations of procedure and specialty.

### Outcome Measures

The primary outcome was a composite of mortality, complications, and readmissions within 30 days. The secondary outcomes were the composite outcome at 90 days and 1 year, individual components of the primary outcome (death, any-cause readmission, and complications) at all time points, and duration of index surgery and length of stay in the hospital up to 30 days.

### Exposure

The exposure was operationalized as the receipt of surgery on Friday the 13th, as compared to surgery on non-Friday the 13th dates (using the flanking Fridays: Friday the 6th and Friday the 20th).

### Covariates

We used multilevel logistic regression models to account for the effects of covariates related to patient, surgeon, anesthesiologist, facility, and treatment characteristics. Patient-related variables included age, sex, comorbidity burden (Johns Hopkins Aggregate Diagnosis Group),^20^ rurality, and socioeconomic status. Physician-related variables included age, sex, specialty, annual case volume (determined by identifying the number of identical procedures the operating surgeon had performed in the previous year, operationalized into quartiles), and years in practice. Anesthesiologist-related variables included age, sex, annual case volume, and years in practice. Facility- and treatment-related variables included facility type (academic *vs* community), admission route (elective *vs* urgent), case complexity, as we previously defined,^15^ and the year and duration of index surgery.

### Statistical Analysis

Using descriptive statistics, we compared the patient, surgeon, anesthesiologist, facility, and treatment characteristics between Friday the 13th and the control groups. Due to the large sample size, we compared groups using standardized differences. A statistically significant standardized difference was defined as greater than 0.10.^21^ We utilized generalized estimating equations with an independent correlation structure, accounting for patient-, surgeon-, anesthesiologist-, and hospital-level covariates, with clustering on surgical procedure to estimate the association between date of surgery and outcomes at 30 days, 90 days, and 1 year from the index surgery.

To estimate adjusted absolute event rates and means, we used models with a Poisson distribution with a log link for binary outcomes and models with a negative binomial with a log link for continuous outcomes, respectively. Models with logit links were used to estimate adjusted relative effects [presented as adjusted odds ratio (aOR) for binary outcomes or adjusted relative risk (aRR) for continuous outcomes]. Estimates were adjusted for the median value of continuous covariates and the third quartile/quintile of categorical covariates. As a sensitivity analysis, we added the duration of the index surgery as a covariate.

We performed subgroup analyses based on predetermined variables to assess the heterogeneity of effect according to patient, surgeon, anesthesiologist, facility, and treatment characteristics. Statistical significance was set at *P* < 0.05 based on a 2-tailed comparison. A Bonferroni correction was used to reduce the risk of making a type I error, utilizing a two-tailed risk level of 0.0083.^22^ All analyses were performed using SAS Enterprise Guide 6.1 (SAS Institute Inc., Cary, NC).

## RESULTS

### Baseline Demographics and Outcomes

Within the study period, there were 24 instances of Friday the 13th, with 48 flanking Fridays used as control. The final cohort included 19,747 patients; 7,349 (37.2%) were in the Friday the 13th group, and 12,398 (62.8%) were in the non-Friday the 13th group (Supplemental Table 1, http://links.lww.com/AOSO/A287). The mean age of surgeons in the study cohort was 48.3 years. Of the studied procedures, 16,330 (82.7%) were elective and 3,417 (17.3%) were urgent. All covariates were balanced between the 2 groups.

### Main Analyses

In the final cohort, the adjusted event rate of the primary outcome of death, readmission, or complications within 30 days was the same in the Friday the 13th group [10.6% (95% CI = 7.9–14.3)] as in the flanking Friday control group [10.3% (95% CI = 7.5–14.1)] (Table 1). At 30 days after index surgery, multivariable regression showed that patients receiving surgery on Friday the 13th were not more likely to experience the composite primary outcome when compared to the flanking Friday control group [aOR = 1.02 (95% CI = 0.94–1.09)] (Table 2). Further, we found no association between receiving surgery on Friday the 13th as compared to flanking Friday control groups with respect to the composite outcome at both 90 days [aOR = 0.97 (95% CI = 0.90–1.04)] and 1 year [aOR = 0.99 (95% CI = 0.94–1.04)] (Table 2). Sensitivity analysis was consistent with the primary analysis for all time points (Supplemental Table 3, http://links.lww.com/AOSO/A290). We found no statistically significant effect for any of the secondary outcomes at 30 days, 90 days, and 1 year.

**TABLE 1.** Adjusted Event Rate of Outcomes within 30- and 90 Days and 1-Year of Index Surgery, by Surgery on Friday the 13^th^ or Flanking Friday

Outcome	Label	Friday the 13^th^ Adjusted Rate or Mean (95% CI)	Flanking Friday Adjusted Rate or Mean (95% CI)
Within 30 days			
Composite endpoint	Rate (%)	10.6 (7.9–14.3)	10.3 (7.5–14.1)
Death	Rate (%)	0.3 (0.1–0.7)	0.3 (0.1–0.7)
Readmission	Rate (%)	5.2 (3.8–7.3)	5.4 (4.2–7.0)
Complications	Rate (%)	5.5 (3.5–8.7)	5.1 (3.1–8.3)
Hospital stay (days)	Mean	3.8 (2.9–5.0)	3.8 (2.9–5.0)
Duration of Index Surgery (hours)	Mean	139.7 (115.4–169.1)	139.2 (114.4–169.2)
Within 90 days			
Composite endpoint	Rate (%)	12.6 (9.7–16.3)	12.7 (9.8–16.5)
Death	Rate (%)	0.7 (0.4–1.4)	0.7 (0.3–1.4)
Readmission	Rate (%)	7.0 (5.6–8.8)	7.5 (6.1–9.1)
Complications	Rate (%)	5.7 (3.6–8.8)	5.3 (3.3–8.6)
Hospital stay (days)	Mean	4.4 (3.3–5.9)	4.6 (3.5–6.1)
Within 1 year			
Composite endpoint	Rate (%)	23.3 (20.3–26.7)	23.4 (20.6–26.6)
Death	Rate (%)	2.1 (1.1–4.1)	2.1 (1.1–3.9)
Readmission	Rate (%)	17.8 (14.4–22.1)	18.3 (14.8–22.7)
Complications	Rate (%)	7.1 (5.0–10.2)	6.7 (4.5–9.9)
Hospital stay (days)	Mean	5.7 (4.3–7.5)	6.1 (4.6–8.0)

**TABLE 2.** Adjusted Risk of Composite, Death, Complications, Readmission and Duration of Surgery at 30- and 90-Days and 1-Year, by Surgery on Friday the 13th or Flanking Friday

	Outcome Within 30 Days		Outcome Within 90 Days		Outcome Within 1 Year	
Outcome	Friday the 13^th^ vs. Flanking Fridays aOR/aRR* (95% CI)	*P* value	Friday the 13^th^ vs. Flanking Fridays aOR/aRR* (95% CI)	P value	Friday the 13^th^ vs. Flanking Fridays aOR/aRR* (95% CI)	*P* value
Composite endpoint	1.02 (0.94–1.09)	0.69	0.97 (0.90–1.04)	0.36	0.99 (0.94–1.04)	0.66
Death	0.99 (0.73–1.34)†	0.96†	1.02 (0.81–1.28)†	0.87†	0.99 (0.84–1.15)	0.86
Re-admission	0.93 (0.80–1.08)	0.33	0.90 (0.81–0.99)	0.04	0.95 (0.89–1.01)	0.12
Complications	1.10 (1.02–1.19)	0.02	1.07 (0.99–1.17)	0.09	1.09 (1.01–1.19)	0.04
Hospital stay (days)	0.99 (0.96–1.02)	0.50	0.96 (0.91–1.01)	0.11	0.94 (0.89–1.00)	0.04
Duration of Index surgery	1.00 (0.99–1.02)	0.92	n.a		n.a	

* Adjusted odds ratio (aOR) for binary outcomes and adjusted relative risk (aRR) for continuous outcomes.

† Results are from ordinary logistic regression modeling, because the GEE model did not converge.

Using GEE modeling dealing with clustering based on procedure fee code (logistic regression with binomial distribution and logit link for binary outcomes, and negative binomial distribution and log link for continuous outcomes), adjusted for surgeon age (continuous), surgeon sex, surgeon annual case volume (quartiles), surgeon specialty, surgeon years of practice (continuous), anesthesiologist age (continuous), anesthesiologist sex, anesthesiologist annual case volume (quartiles), anesthesiologist years of practice (continuous), patient age (continuous), patient sex, patient comorbidity (categorical), rurality (rural vs. urban), income quintile, Local Health Integrated Network, hospital status (academic vs. community), and index year.

### Subgroup Analyses

**FIGURE 2.**
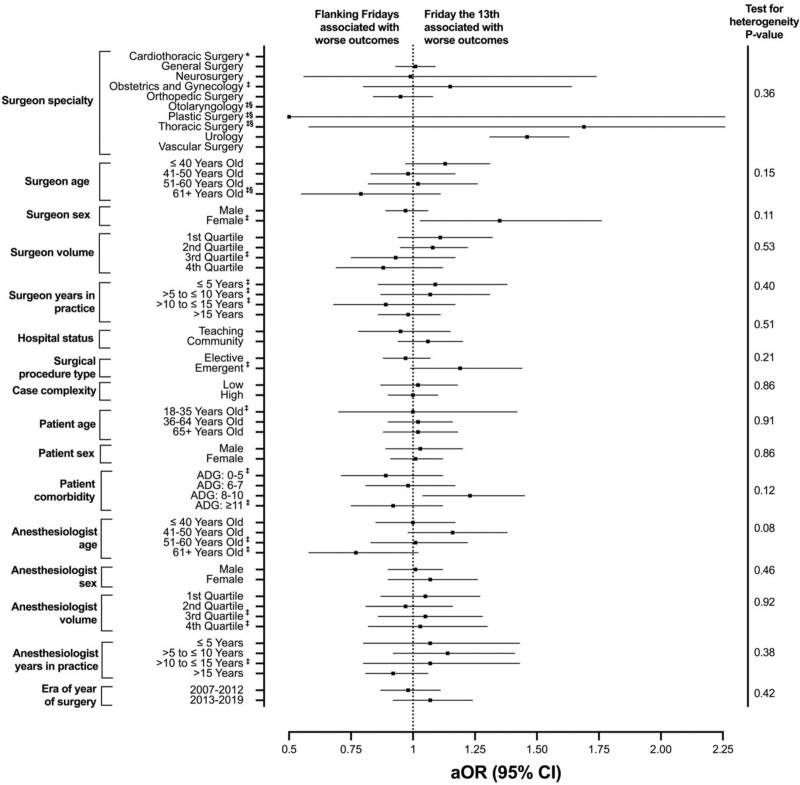
Likelihood of adverse postoperative outcomes within 30 days of Friday type of surgery, stratified by Surgeon, Facility, Procedure, Patient, and Anesthesiologist Factors. Note: using GEE modeling dealing with clustering based on procedure fee code (logistic regression with binomial distribution and logit link), adjusted for surgeon age (continuous), surgeon sex, surgeon annual case volume (quartiles), surgeon specialty, surgeon years of practice (continuous), anesthesiologist age (continuous), anesthesiologist sex, anesthesiologist annual case volume (quartiles), anesthesiologist years of practice (continuous), patient age (continuous), patient sex, patient comorbidity (categorical), rurality (rural *vs* urban), income quintile (quintiles), Local Health Integrated Network, hospital status (academic *vs* community), and index year, whereas the variable in the subgroup was excluded. ‡Results for the analysis were from an ordinary logistic regression, because the GEE modeling did not converge. §Model is questionable. *Maximum likelihood estimate does not exist due to small sample size. GEE indicates generalized estimating equations.

In subgroup analyses utilizing the primary composite outcome at 30 days (Fig. 2), there was no evidence of effect modification when stratified by patient age, patient sex, patient comorbidity, surgeon specialty, surgeon age, surgeon sex, surgeon volume, surgeon years in practice, anesthesiologist age, anesthesiologist sex, anesthesiologist volume, anesthesiologist years in practice, hospital status, and surgical procedure type (elective *vs* emergent). Further, we assessed the heterogeneity of effect for the association between Friday the 13th *versus* flanking Fridays and outcomes for patients undergoing high *versus* low complexity procedures. We found that the rates of high-complexity surgery were comparable on Friday the 13th (65.5%) and flanking Fridays (64.9%) (Supplemental Table 1, http://links.lww.com/AOSO/A287). We found patients undergoing surgery on Friday the 13th were not at an increased risk of adverse outcomes when they underwent low [aOR = 1.02 (95% CI = 0.87–1.18)] or high [aOR = 1.00 (95% CI = 0.90–1.10)] complexity procedures (Fig. 2).

## DISCUSSION

In this retrospective, population-based analysis, patients undergoing surgery on Friday the 13th were not at increased likelihood of adverse surgical outcomes. This finding was robust across numerous subgroups. These results should be reassuring for patients, surgeons, and policymakers because surgical care is unlikely to be influenced by the psychological effects of receiving a surgical procedure on Friday the 13th.

Although medicine is in large part considered to be evidence-based and driven by logic, historical traditions and beliefs contribute substantially to its practice. As a result, superstitious notions are pervasive in medicine, the foremost of which may be the fear that remarking on a “quiet” or “slow” shift is doomed to jinx or reverse the good luck.^23,24^ Further, despite data to the contrary,^25,26^ there remains a common belief that some physicians and nurses are more or less likely to have a large volume or complexity of work, followed by a so-called black cloud or white cloud, respectively. In the context of surgery, many surgeons have their own small habits and rituals that underlie their practices. For example, many insist on starting the day with a specific song in the operating room or wearing a “lucky” scrub cap. Such practices and customs reiterate the subtle ways in which physicians buy into superstition, maintaining their sacred ceremonies and protocols as a means of reducing variation and quelling anxiety.^1^

Though the rational mind can appreciate that there is no cogent or tangible mechanism to drive a Friday the 13th effect, the power of perception in influencing behavior should not be readily dismissed. Particularly in the field of surgery, there are often numerous complex and uncontrollable factors at play that contribute to a degree of uncertainty and unpredictability with each procedure. The potential for any real or perceived force to perturb a seemingly delicate universal balance could be enough to change behavior and negatively impact outcomes. Physicians may behave differently on a day they associate with bad luck than they would on other days, altering their regular routines and making decisions based, consciously or subconsciously, out of fear. The extensiveness of studies into the effects of superstition on medicine, analyzing everything from lunar phases to zodiac signs, is further evidence of the prevalence of magical thinking within the field of medicine.^14^

In the present study, we found that overall, the Friday the 13th superstition does not affect surgical outcomes to a clinically significant degree. Despite the widespread presence of superstitious beliefs in medicine, fears of entering the operating room on Friday the 13th appear to be unfounded. We hope that the presented evidence can help to alleviate apprehension for both physicians and patients.

Our study has limitations. First, because the only day of the week analyzed in this study is Friday, these results may not be broadly generalizable, as there may be other factors that influence postoperative outcomes for surgery performed at the end of the week. In a separate analysis, we have identified that, not unsurprisingly, junior surgeons, as defined by years of experience, are more likely to operate on Fridays than Mondays (unpublished data). Therefore, it follows that our analyses of Friday the 13th outcomes *versus* flanking Fridays may not be generalizable to all surgeons, and rather are findings restricted specifically to more junior surgeons, who are more likely to operate on Friday. Subgroup analysis by case complexity showed no differences between the 2 groups, but there may be a more complex subset of cases within each procedure that are not typically performed on Fridays and therefore unaccounted for in our study. Second, surgeons and patients, particularly those who are more superstitious, may be less likely to book elective surgeries on Friday the 13th, leading to inherent bias in the cases analyzed in the present study. Nevertheless, we found no evidence of effect modification when considering the urgency of the surgical procedure (elective *vs* emergent). Third, these results may not be generalizable to patients receiving surgeries in other countries where Friday the 13th superstition is less prevalent and may not be applicable to similarly unlucky days or events.

The results of our study show that surgical patients on Friday the 13th are not at increased likelihood of adverse surgical outcomes. Further studies are necessary to better understand the nuances of how Friday the 13th superstition impacts surgical practice, specifically with respect to cultural and ideological beliefs in different practice settings. For now, physicians and patients can rest assured. If you must go to the operating room on Friday the 13th, it is still very safe.

## ACKNOWLEDGEMENTS

All authors contributed to the composition of this article.

## Supplementary Material






