# Supplementary material for: Superstition in Surgery: A Population-Based Cohort Study to Assess the Association Between Surgery on Friday the 13th and Postoperative Outcomes
Source: Ann Surg Open. 2024 Feb 12;5(1):e375. doi: 10.1097/AS9.0000000000000375 (PMC11175938; doi:10.1097/AS9.0000000000000375)
Supplement: Supplementary file 1 [file as9-5-e375-s001.pdf]

Supplementary Table 2. Included procedures and corresponding OHIP fee codes

	Surgery	OHIP Fee Code
A.	Coronary artery bypass grafting	R742, R743
B.	Femoral-popliteal bypass	R791, R794
C.	Abdominal aortic aneurysm repair	R802, R817, R877
D.	Appendectomy	S205
E.	Cholecystectomy	S287
F.	Gastric bypass Roux-en-Y	S120
G.	Colon resection	S166, S167, S168, S169, S170, S171, S173, S174, S213, S214, S215, S216, S217
H.	Liver resection	S267, S269, S270, S271, S275
I.	Hysterectomy	S757, S816, S758, S759, S710, S763
J.	Spinal surgery	
i.	Anterior decompression	N500, N501, N502, N503, N504, N505, N506, N507, N508, N579
ii.	Anterior arthrodesis	N516, N517, N518, N559, N580
iii.	Posterior decompression	N509, N510, N520, N511, N512, N524
iv.	Posterior arthrodesis	N515, N519, N514, N581, N582, N533
K.	Craniotomy for brain tumor/biopsy	N102, N103, N151, N152, N153 / N113
L.	Knee replacement TKR	R441
M.	Hip replacement THR	R440
N.	Open repair femoral neck/femoral shaft fracture	F099, F100, F101/F096
O.	Total thyroidectomy	S788
P.	Neck dissection	R910, R915
Q.	Lung resection pneumonectomy, lobectomy or segmental resection	M142, M143, M144
R.	Radical cystectomy	S440, S453
S.	Radical prostatectomy	S651
T.	Transurethral resection of prostate	S655
U.	Carpal tunnel release	N290
V.	Breast reduction	R110
