# Supplementary material for: Superstition in Surgery: A Population-Based Cohort Study to Assess the Association Between Surgery on Friday the 13th and Postoperative Outcomes
Source: Ann Surg Open. 2024 Feb 12;5(1):e375. doi: 10.1097/AS9.0000000000000375 (PMC11175938; doi:10.1097/AS9.0000000000000375)
Supplement: Supplementary file 2 [file as9-5-e375-s002.pdf]

Supplemental Table 1: Baseline Characteristics of Study Cohort, by Surgery on Friday the 13th or Flanking Friday cohort

Variable	Label or value	Friday the 13th	Flanking Friday	Total	Standardized Difference
		N=7,349	N=12,398	N=19,747	
**Surgeon characteristics**					
Age	Mean (SD), years	48.0 (9.6)	48.4 (9.6)	48.3 (9.6)	0.04
Sex, n (%)	Female	1,201 (16.3%)	1,857 (15.0%)	3,058 (15.5%)	0.038
Annual case volume (quartiles), n (%)	1 - Lowest	1,838 (25.0%)	3,069 (24.8%)	4,907 (24.8%)	0.006
	2	1,846 (25.1%)	3,169 (25.6%)	5,015 (25.4%)	0.01
	3	1,753 (23.9%)	2,953 (23.8%)	4,706 (23.8%)	0.001
	4 - Highest	1,912 (26.0%)	3,207 (25.9%)	5,119 (25.9%)	0.003
Years in practice	Mean (SD), years	14.5 (8.8)	15.0 (8.7)	14.8 (8.7)	0.055
Specialty, n (%)	Cardiothoracic Surgery	23 (0.3%)	26 (0.2%)	49 (0.2%)	0.020
	General Surgery	2,556 (34.8%)	4,120 (33.2%)	6,676 (33.8%)	0.033
	Neurosurgery	418 (5.7%)	632 (5.1%)	1,050 (5.3%)	0.026
	Obstetrics and Gynaecology	987 (13.4%)	1,662 (13.4%)	2,649 (13.4%)	0.001
	Orthopaedic Surgery	2,308 (31.4%)	4,034 (32.5%)	6,342 (32.1%)	0.024
	Otolaryngology	129 (1.8%)	205 (1.7%)	334 (1.7%)	0.008
	Plastic Surgery	318 (4.3%)	551 (4.4%)	869 (4.4%)	0.006
	Thoracic Surgery	88 (1.2%)	187 (1.5%)	275 (1.4%)	0.027
	Urology	500 (6.8%)	947 (7.6%)	1,447 (7.3%)	0.032
	Vascular Surgery	22 (0.3%)	34 (0.3%)	56 (0.3%)	0.005
**Anesthesiologist characteristics**					
Age	Mean (SD), years	48.9 (10.3)	48.8 (10.2)	48.8 (10.3)	0.009
Sex, n (%)	Female	2,023 (27.5%)	3,257 (26.3%)	5,280 (26.7%)	0.028
Annual case volume (quartiles), n (%)	1 - Lowest	1,784 (24.3%)	2,816 (22.7%)	4,600 (23.3%)	0.037
	2	1,850 (25.2%)	3,297 (26.6%)	5,147 (26.1%)	0.032
	3	1,837 (25.0%)	3,151 (25.4%)	4,988 (25.3%)	0.010
	4 - Highest	1,878 (25.6%)	3,134 (25.3%)	5,012 (25.4%)	0.006
Years in practice	Mean (SD), years	14.7 (9.5)	14.6 (9.4)	14.6 (9.5)	0.013
**Patient characteristics**					
Age	Mean (SD), years	58.1 (17.1)	58.5 (17.1)	58.3 (17.1)	0.022
Sex, n (%)	Female	4,608 (62.7%)	7,713 (62.2%)	12,321 (62.4%)	0.010
Comorbidity, n (%)	ADG 0-5	1,897 (25.8%)	3,201 (25.8%)	5,098 (25.8%)	0.000
	ADG 6-7	1,783 (24.3%)	3,010 (24.3%)	4,793 (24.3%)	0.000
	ADG 8-10	2,239 (30.5%)	3,698 (29.8%)	5,937 (30.1%)	0.014
	AGD>=11	1,430 (19.5%)	2,489 (20.1%)	3,919 (19.8%)	0.016

Rurality, n (%)	Urban	6,483 (88.2%)	10,972 (88.5%)	17,455 (88.4%)	0.009
	Rural	866 (11.8%)	1,426 (11.5%)	2,292 (11.6%)	0.009
Income quintile, n (%)	1 - Lowest	1,349 (18.4%)	2,382 (19.2%)	3,731 (18.9%)	0.022
	2	1,444 (19.6%)	2,487 (20.1%)	3,931 (19.9%)	0.010
	3	1,559 (21.2%)	2,555 (20.6%)	4,114 (20.8%)	0.015
	4	1,531 (20.8%)	2,490 (20.1%)	4,021 (20.4%)	0.019
	5 - Highest	1,466 (19.9%)	2,484 (20.0%)	3,950 (20.0%)	0.002
**Other characteristics**					
Hospital status, n (%)	Community hospital	4,785 (65.1%)	8,222 (66.3%)	13,007 (65.9%)	0.025
	Academic hospital	2,564 (34.9%)	4,176 (33.7%)	6,740 (34.1%)	0.025
Surgical procedure type, n (%)	Elective	6,116 (83.2%)	10,214 (82.4%)	16,330 (82.7%)	0.022
	Urgent	1,233 (16.8%)	2,184 (17.6%)	3,417 (17.3%)	0.022
Case complexity, n (%)	Low	2,539 (34.5%)	4,355 (35.1%)	6,894 (34.9%)	0.012
	High	4,810 (65.5%)	8,043 (64.9%)	12,853 (65.1%)	0.012
Duration of index surgery	Missing on duration, n (%)	377 (5.1%)	666 (5.4%)	1,043 (5.3%)	0.011
	Non-missing on duration, n (%)	6,972 (94.9%)	11,732 (94.6%)	18,704 (94.7%)	0.011
	Mean (SD), minutes	125.1 (76.4)	124.0 (118.1)	124.4 (104.5)	0.011
	Median (IQR), minutes	106 (76-152)	105 (75-150)	106 (75-150)	0.029

SD: standard deviation, IQR: interquartile range
