# Supplementary material for: Superstition in Surgery: A Population-Based Cohort Study to Assess the Association Between Surgery on Friday the 13th and Postoperative Outcomes
Source: Ann Surg Open. 2024 Feb 12;5(1):e375. doi: 10.1097/AS9.0000000000000375 (PMC11175938; doi:10.1097/AS9.0000000000000375)
Supplement: Supplementary file 3 [file as9-5-e375-s003.pdf]

Supplementary Table 3. Sensitivity Analysis: Multivariable Generalized Estimating Equation Regression Models, with Clustering based on Procedure Fee Code for Outcomes within 30- and 90-Days and 1-Year of Index Surgery, by Surgery on Friday the 13th or Flanking Friday

	**Outcome within 30 Days**		**Outcome within 90 Days**		**Outcome within 1 Year**	
**Outcome**	**Friday the 13th vs. Flanking Fridays aOR/aRR (95% CI)**	**P value**	**Friday the 13th vs. Flanking Fridays aOR/aRR (95% CI)**	**P value**	**Friday the 13th vs. Flanking Fridays aOR/aRR (95% CI)**	**P value**
Composite Endpoint	0.98 (0.91-1.06)	0.64	0.95 (0.89-1.02)	0.13	0.98 (0.94-1.02)	0.40

* Adjusted odds ratio (aOR) for binary outcomes and adjusted relative risk (aRR) for continuous outcomes.

Note: using GEE modeling dealing with clustering based on procedure fee code (logistic regression with binomial distribution and logit link), adjusted for surgeon age (continuous), surgeon sex, surgeon annual case volume (quartiles), surgeon specialty, surgeon years of practice (continuous), anesthesiologist age (continuous), anesthesiologist sex, anesthesiologist annual case volume (quartiles), anesthesiologist years of practice (continuous), patient age (continuous), patient sex, patient comorbidity (categorical), rurality (rural vs. urban), income quintile, LHIN, hospital status (academic vs. community), and index year, **as well as duration of index surgery.**
